# Good response with nivolumab and cabozantinib combination therapy in patients with metastatic collecting duct carcinoma with high expression of PD-L1, c-MET, and AXL

**DOI:** 10.1007/s13730-025-01027-9

**Published:** 2025-09-08

**Authors:** Tatsuya Umemoto, Masanori Hasegawa, Jun Naruse, Tatsuo Kano, Nobuyuki Nakajima, Masahiro Nitta, Yoshiaki Kawamura, Hiroshi Kajiwara, Sunao Shoji

**Affiliations:** 1 Department of Urology, Tokai University School of Medicine, 143 Shimokasuya, Isehara, Kanagawa 259-1193 Japan; 2 Department of Pathology, Tokai University School of Medicine, 143 Shimokasuya, Isehara, Kanagawa 259-1193 Japan

**Keywords:** Collecting duct carcinoma, Nivolumab, Cabozantinib, Renal cell carcinoma

## Abstract

Collecting duct carcinoma (CDC) is a rare subtype of renal cell carcinoma with a poor prognosis. Moreover, despite various chemotherapeutic strategies and administration of several tyrosine kinase inhibitors for metastatic CDC, the outcomes remain unfavorable, with no established treatment. Herein, we report the cases of two patients with CDC who exhibited a good response to nivolumab and cabozantinib combination therapy. Both patients were diagnosed with CDC via a needle biopsy of the renal tumor, revealing high expression levels of programmed death ligand 1 (PD-L1), c-MET, and AXL. After 10 and 12 courses of combination therapy for Cases 1 and 2, respectively, significant response was observed against the primary and metastatic lesions. Subsequently, the patients underwent laparoscopic nephrectomy. To the best of our knowledge, this is the first report documenting the favorable therapeutic response of nivolumab and cabozantinib combination therapy against metastatic CDC in patients with high expression of the corresponding molecular targets. These findings may have a strong implication in the selection of first-line systemic therapies for metastatic CDC.

## Introduction

Collecting duct carcinoma (CDC) is a rare subtype of renal cell carcinoma, observed in < 1% of all renal cell carcinoma (RCC) cases. It is generally diagnosed at an advanced stage characterized by rapid progression and poor prognosis [1, 2]. However, despite the utilization of various chemotherapeutic strategies and administration of several tyrosine kinase inhibitors for CDC, responses to these interventions remain unfavorable. Furthermore, no established treatment is currently available [3]. Recent studies have revealed an increased expression of programmed death ligand 1 (PD-L1) in CDC cells and reported partial efficacy of immune checkpoint inhibitors, such as anti-programmed death 1 (PD-1) and cytotoxic T-lymphocyte antigen 4, in CDC treatment [4, 5]. In contrast, nivolumab and cabozantinib combination therapy is the first-line systemic therapy for metastatic clear cell RCC which induced a significantly prolonged overall survival in a phase III clinical trial compared to that of sunitinib monotherapy [6]. However, the use of nivolumab and cabozantinib combination therapy in CDC lacks documentation. To address this gap, herein, we present two cases of patients with metastatic CDC expressing high levels of PD-L1, c-MET, and AXEL, who exhibited favorable responses to nivolumab and cabozantinib combination therapy.

## Case report

### Case 1

**Fig. 1:**
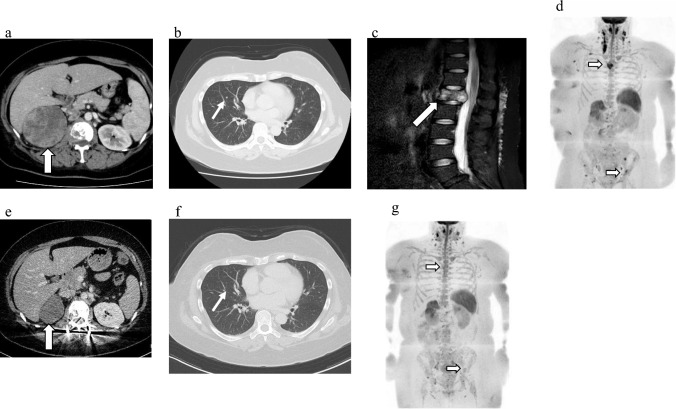
Imaging examinations of case 1 before (**a**–**d**) and after (**e**–**g**) combined nivolumab and cabozantinib treatment. **a** Compute tomography (CT) showing primary right renal tumor before combined treatment. **b** CT showing lung metastatic lesion before combined treatment. **c** Magnetic resonance imaging showing L1 bone metastatic lesion before combined treatment. **d** Diffusion-weighted imaging with background suppression (DWIBS) showing metastatic lesions in the sternum and left ilium before combined treatment. **e **CT showing primary right renal tumor after combined treatment. **f** CT showing lung metastatic lesion after combined treatment. **g** DWIBS showing metastatic lesions in the sternum and left ilium after combined treatment

**Fig. 2:**
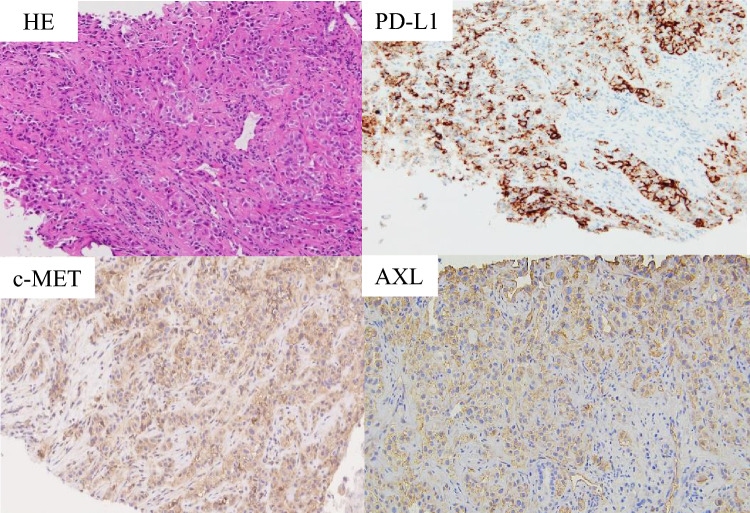
Pathological examination of case 1. Hematoxylin and eosin staining and PD-L1 (antibody; ab205921, Abcam), c-MET (antibody; ab51067, Abcam), and AXL (antibody; AF154, R&D Systems) expressions in biopsy specimens (original magnification × 200)

A 41-year-old woman presented to our department with gross hematuria, right lower back pain, and a right abdominal mass. Computed tomography (CT) revealed a 9-cm right renal tumor, multiple lung metastases, including an L1 lumbar metastasis causing spinal cord compression (Fig. 1a–c). Diffusion-weighted whole-body magnetic resonance imaging (MRI) with background body signal suppression (DWIBS) revealed additional bone metastases in the sternum and left ilium (Fig. 1d). The International Metastatic RCC Database Consortium risk score was 6 points; thus, the patient was diagnosed with poor-risk metastatic RCC. Consequently, a needle biopsy of the renal tumor was performed and the tumor was diagnosed as CDC based on immunohistochemical analysis showing the following expression pattern: CK7( +), CK20(–), vimentin( +), p63( +), uroplakin III(–), Pax8( +), Pax2(–). In addition, immunostaining with an anti-PD-L1 antibody revealed high PD-L1 expression, with a tumor proportion score (TPS) > 80% (Fig. 2). Furthermore, immunohistochemistry (IHC) scores > 2 for both c-MET and AXL were observed in 70% and 90% of tumor specimens, respectively (Fig. 2).

Following diagnosis, balloon kyphoplasty and percutaneous pedicle screw fixation were performed for the L1 metastasis to prevent spinal fractures and spinal cord injuries. Postoperatively, combination therapy with 240 mg nivolumab every 2 weeks and oral 40 mg cabozantinib once a day was introduced as the first-line systemic treatment for CDC. After two courses of nivolumab and cabozantinib combination therapy, the lung metastasis disappeared (Fig. 1e). However, after six courses of combination therapy, the patient developed Grade-3 diarrhea, according to the Common Terminology Criteria for Adverse Events. This was suggested to be an adverse event due to cabozantinib therapy because the symptoms improved after a temporary cabozantinib interruption. Cabozantinib administration was resumed at 20 mg after the symptoms resolved and later increased to 40 mg. After 10 months of systemic treatment with 12 courses, CT showed a significant reduction in the size of the renal tumor, and DWIBS revealed decreased tumor activity in terms of bone metastases (Fig. 1f, g). Subsequently, a laparoscopic radical nephrectomy was performed. More than 90% of the tumor cells showed necrosis and fibrosis. The patient did not receive any medication postoperatively and exhibited no tumor recurrence for 6 months.

### Case 2

**Fig. 3:**
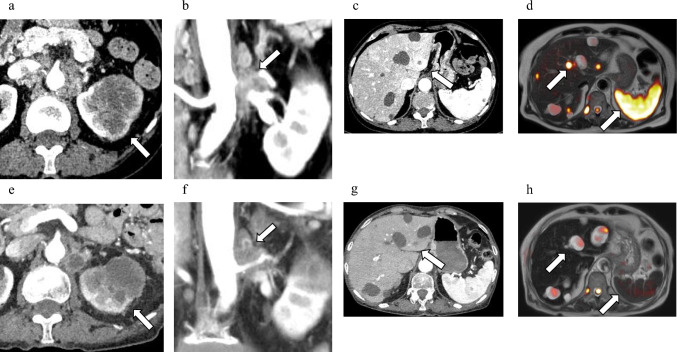
Imaging examinations of Case 2 before and after combined nivolumab and cabozantinib treatment. **a** Compute tomography (CT) showing primary left renal tumor before combined treatment. **b** CT showing para-aortic lymph node metastatic lesions before combined treatment. **c** CT showing liver metastatic lesion before combined treatment. **d** Diffusion-weighted imaging with background suppression (DWIBS) showing liver and splenic metastatic lesions before combined treatment. **e** CT showing the primary left renal tumor after combined treatment. **f** CT showing para-aortic lymph node metastatic lesions after combined treatment. **g** CT showing liver metastatic lesions after combined treatment. **h** DWIBS showing liver and splenic metastatic lesions after combined treatment

**Fig. 4:**
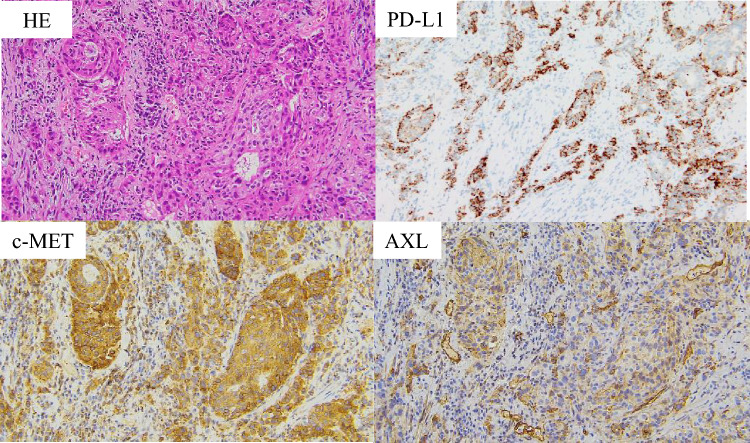
Pathological examination of Case 2. Hematoxylin and eosin staining and PD-L1 (antibody; ab205921, Abcam, Cambridge, UK), c-MET (antibody; ab51067, Abcam), and AXL (antibody; AF154, R&D Systems Minneapolis, MN, USA) expressions in biopsy specimens (original magnification, × 200)

A 76-year-old woman with a left renal tumor and multiple liver masses was referred to our department. CT and DWIBS revealed a 60-mm renal tumor with a renal venous tumor thrombus, multiple para-aortic lymph node metastases, and multiple liver and splenic metastases (Fig. 3a–d). Renal tumor biopsy confirmed the diagnosis of CDC. In addition, high expression levels of PD-L1 (TPS; 70%), c-MET (IHC > 2; 100%), and AXL (IHC > 2; 70%) were observed in the biopsy specimens (Fig. 4).

**Fig. 5:**
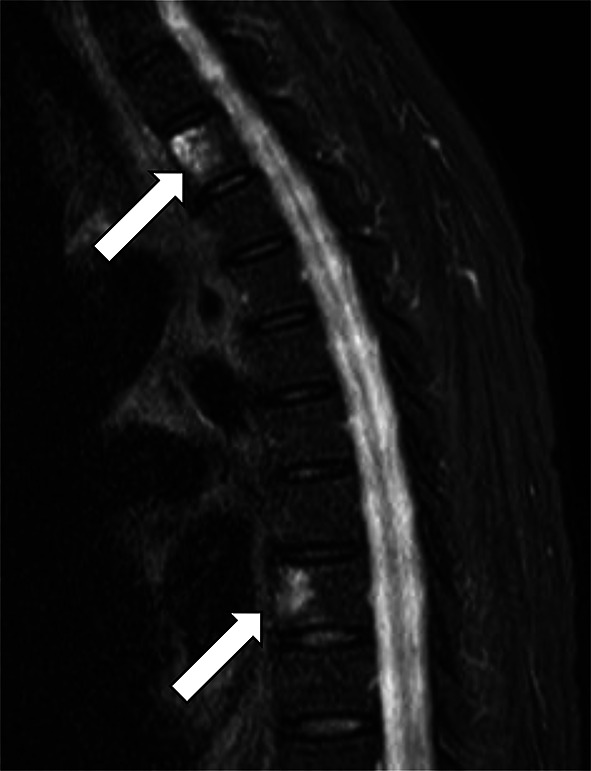
DWIBS showing bone metastasis of Th2 and 8 at 5 months after starting systemic treatment

Consequently, nivolumab and cabozantinib combination therapy was initiated at 240 mg every 2 weeks and 40 mg orally once a day, respectively. However, during the first combination therapy, the patient experienced multiple brain strokes, attributed to Trousseau’s syndrome caused by CDC, leading to temporary suspension of RCC therapy. The patient received anticoagulation therapy for stroke including heparin calcium, and resumed combination therapy for RCC after 2 weeks of interruption. However, after two cycles, the patient developed immune-related thrombocytopenia and immunotherapy was discontinued again. Treatment with 1mg/kg prednisone was initiated with a step taper. Subsequently, cabozantinib monotherapy was initiated, with metastatic lesions of the liver and spleen showing a complete response on DWIBS after 4 months (Fig. 3e–h). Unfortunately, DWIBS performed 5 months after starting systemic treatment revealed new bone metastasis in Th 2 and 8 (Fig. 5). Consequently, the patient underwent stereotactic body radiation therapy (SBRT; 27 Gy in three fractions) for each vertebra. Embracing a multidisciplinary approach for treating advanced cancer including CDC, robot-assisted radical nephrectomy, and para-aortic lymph node dissection were performed 7 months after starting treatment. Tumor necrosis was observed in approximately 60% of the renal tumor and para-aortic lymph node metastases. The postoperative course was uneventful, without any new metastatic lesions developing for 3 months after operation, and cabozantinib monotherapy was continued.

## Discussion

We encountered two cases of metastatic CDC exhibiting a favorable response to nivolumab and cabozantinib combination therapy and high expression of PD-L1, c-MET, and AXL. The high PD-L1 expression in both the cases suggests a potential association between higher expression of PD-L1 and the efficacy of nivolumab, consistent with the finding of previous case reports [4, 5]. In contrast, although cabozantinib monotherapy is reportedly effective for treating CDC eliciting a partial response [7], immunostaining of the tumor has not been performed previously, and the biological evidence remains unclear. In both of our cases, c-MET and AXL, in addition to PD-L1, were highly expressed in the biopsy specimens. These findings are of great interest when considering systemic CDC therapy because they are the major inhibitory targets of cabozantinib. To summarize, nivolumab, targeting PD-1, and cabozantinib, targeting c-MET and AXL, may be considered a promising first-choice treatment option for metastatic CDC. However, it should be noted that the correlation between the expression of c-MET and AXL in CDC and the therapeutic effect of cabozantinib remains unclear.

The main target molecules of cabozantinib, which was used to treat CDC in this case, are c-MET and AXL, which are strongly implicated in the pathogenesis of clear cell renal carcinoma. However, the expression and relevance of c-MET and AXL in CDC pathogenesis remain unclear. In the present study, immunohistochemical staining in these two CDC cases confirmed the expression of both AXL and c-MET. This finding is notable, as it suggests a relationship between c-MET or AXL and the pathogenesis of CDC. In cases 1 and 2, PD-L1 and c-MET expression remained detectable in the resected specimens posttreatment compared with the pretreatment biopsy specimens. Although AXL expression remained present in case 1, it was decreased, whereas it was no longer detectable in case 2 (data not shown). It is difficult to determine whether the difference in AXL expression ultimately influenced the late treatment outcome; however, the development of bone metastasis in case 2 suggests that the loss of AXL expression may have limited the efficacy of cabozantinib.

DWIBS is a whole-body MRI method, first reported by Takahara et al. in 2004 [8]. The advantages of DWIBS include its ability to evaluate the whole body without radiation exposure and to quantify the total tumor volume, facilitating assessment of treatment response. However, to date, the diagnostic performance of DWIBS in renal cell carcinoma, especially in CDC, has not yet been fully validated. In the present study, the application of DWIBS in the two cases enabled the detection of CDC metastases and monitoring treatment-related changes, suggesting its effectiveness. In addition, DWIBS has demonstrated effectiveness in detecting oligo-bone metastasis in prostate cancer [9] similarly observed in Case 2 in our study. However, its disadvantages include difficulty in detecting lung metastases and the possibility of false positives due to inflammatory changes. Although sufficient evidence regarding the detection power or advantages of DWIBS compared to conventional diagnostic methods, such as CT, is currently lacking, DWIBS combined with CT is expected to be a useful imaging method for RCC management.

SBRT was performed for bone metastasis in Case 2 as part of the multidisciplinary treatment. Onal et al. previously reported a 1‑year local control rate of 94.9% for SBRT in bone oligometastasis of RCC [10]. However, they also noted disease progression in untreated regions following SBRT in nearly half of the patients and suggested the inclusion of an additive effective systemic treatment to improve treatment outcomes. In Case 2, the new bone lesion appeared while the primary tumor and para-aortic lymph node metastasis were present, indicating that it does not strictly fulfill the definition of oligometastasis. Furthermore, the short observation period in this case was insufficient to fully evaluate the treatment effect of SBRT; therefore, longer follow-up is required.

To the best of our knowledge, this is the first report demonstrating the efficacy of nivolumab and cabozantinib combination therapy in CDC expressing c-MET, AXL, and PD-L1. Our findings underscore the significance of c-MET and AXL as major tumor growth pathways in CDC. Although our study revealed promising results, we acknowledge the relatively short follow-up period. A longer follow-up is necessary to assess disease progression and recurrence. Therefore, further studies with larger sample sizes and longer follow-up are needed to confirm the durability of treatment responses.

## Data Availability

The datasets generated and/or analyzed during the current study are not publicly available because the data also form part of an ongoing study but are available from the corresponding author upon reasonable request.
